# COVID-19–Related Social Isolation, Self-Control, and Internet Gaming Disorder Among Chinese University Students: Cross-Sectional Survey

**DOI:** 10.2196/52978

**Published:** 2024-09-10

**Authors:** Yufang Guo, Fangyan Yue, Xiangyu Lu, Fengye Sun, Meixing Pan, Yannan Jia

**Affiliations:** 1 School of Nursing and Rehabilitation Shandong University Jinan China; 2 Hebei Provincial Mental Health Center Baoding China; 3 Committee of The Communist Youth League Shandong University Jinan China

**Keywords:** COVID-19 pandemic, internet gaming disorder, self-control, social isolation, university students, game, gaming, games, addict, addictive, addiction, addictions, university, universities, college, colleges, postsecondary, higher education, student, students, China, Chinese, isolation, isolated, self-compassion, mental health, association, associations, correlation, causal, correlated, correlations

## Abstract

**Background:**

Internet gaming disorder among university students has become a great concern for university counsellors worldwide since the COVID-19 pandemic. The factors influencing the development of internet gaming disorder in students during the COVID-19 pandemic could be different from those before the pandemic.

**Objective:**

This study aims to explore the associations among social isolation, self-control, and internet gaming disorder in Chinese university students and to examine whether self-control mediates the positive effects of social isolation on internet gaming disorder.

**Methods:**

A cross-sectional survey was employed to collect data from university students in Shandong province of China from April to September 2022. The Isolation subscale of the Self-Compassion Scale, Self-Control Scale, and Internet Gaming Disorder Scale were used to assess the social isolation, self-control, and internet gaming disorder among university students, respectively. Models 4 and 5 of PROCESS software were used to analyze the mediating role of self-control and the moderating role of gender on the association between social isolation and internet gaming disorder.

**Results:**

A total of 479 students were recruited from 6 universities located in 3 different regions of Shandong, China. Students had low levels of internet gaming disorder and moderate levels of social isolation and self-control, with mean scores of 8.94 (SD 9.06), 12.04 (SD 3.53), and 57.15 (SD 8.44), respectively. Social isolation was positively correlated with internet gaming disorder (*r*=0.217; *P*<.001), and self-control was negatively correlated with social isolation (*r*=–0.355; *P*<.001) and internet gaming disorder (*r*=–0.260; *P*<.001). Self-control played a mediating role in the association between social isolation and internet gaming disorder (β=–.185, 95% CI –.295 to –.087). The effects of social isolation on internet gaming disorder among female students were lower than those among male students.

**Conclusions:**

Self-control was a mediator in the association between social isolation and internet gaming disorder. Moreover, gender played a moderating role in the association between social isolation and internet gaming disorder. This study highlights the need to alleviate the development of internet gaming disorder among students during a pandemic, especially that of male students. Effective interventions that lessen social isolation and promote self-control should be developed.

## Introduction

The COVID-19 pandemic was declared a global health emergency by the World Health Organization in March 2020, which significantly impacted educational training institutions worldwide [1]. Previous studies have shown that because of educational interruptions, remote learning, less physical activity, financial concerns, social isolation, lack of belongingness, and the rapid rise in the number of COVID-19 infections, university students were one of the most vulnerable groups to experience anxiety, depression, frustration, loneliness, substance misuse, and addiction [2-4]. To alleviate the negative impacts of COVID-19 on mental and physical health, university students choose internet gaming as the main source of entertainment [5]. According to the research report on the internet usage of minors in China in 2023 released by China Internet Network Information Center, 522 million Chinese citizens often played internet games, and university students are one of the main groups who often play internet games [6,7].

Internet gaming disorder (IGD), classified as an addictive disorder by the *Diagnostic and Statistical Manual of Mental Disorders, fifth edition* (*DSM-5*) and the *eleventh revision of the International Classification of Diseases* (*ICD-11*), is defined as a pattern of continuous or repeated gaming behavior, manifested through 5 of the 9 diagnosis criteria in the *DSM-5* (withdrawal, loss of control, loss of interest, preoccupation or obsession, tolerance, deceiving, continued overuse, functional impairment, and escape of negative feelings) or diagnosis in the *ICD-11* (impaired control of the game, increasing priority for gaming, continuous or upgraded gaming despite negative outcomes) [8,9]. Son et al [10] found that 36.4% of the university students played internet games 3-4 times per week, 19.5% played internet games daily, and 48.1% played an internet game for over 2 hours. Vahidi et al [11] found that 48.5% of the university students played internet games, and among them, 4.3% were diagnosed with IGD. Siste et al [12] found that the prevalence rate of IGD was 2.03% among university students, and students with IGD played games 19 hours per week. Previous evidence shows that IGD leads to maladjustments in students’ daily life, such as headaches, attention-deficit/hyperactivity disorder, alexithymia, increased aggression, poor psychological well-being, and a decline in study achievement [13-15]. IGD also inclines students to flee their homes and engage in substance abuse, self-harm, and even suicidality when it becomes severe [16,17]. Therefore, exploring the influencing factors for IGD in university students during the COVID-19 pandemic is crucial for university counsellors and students themselves to take effective interventions to treat and relieve symptoms of IGD and prevent IGD in future pandemics.

Several qualitative and quantitative studies have indicated that social isolation plays a key role in the emergence of IGD, especially as seen during the COVID-19 pandemic [18,19]. Social isolation is an objective measure reflecting an individual’s absence or limitation of social contact or interactions with friends, family, neighbors, and colleagues [20]. Social isolation causes profound changes in social relationships, which are linked with loneliness, anxiety, depression, and exhaustion. It is possible that individuals who feel social isolation are more likely to surf online to address their loneliness [21,22]. Moreno et al [23] reported that because of poor quality and infrequent social interactions, the prevalence of IGD among young adults was 4.43% during the COVID-19 pandemic. Gaming addiction is referred to as a consequence of dealing with COVID-19–related psychological distress and social isolation [24]. According to previous studies, social isolation has damaging effects on the IGD of university students influenced by personality characteristics [25,26]. However, the critical factors and impact pathway between social isolation and IGD remain unclear and need further investigation.

Self-control refers to individuals’ ability to regulate their thoughts, feelings, and actions, when permanently valued purposes collide with momentarily more satisfying goals [27]. As a facet of personality traits, self-control can involve engaging in desired behaviors and deserting undesired ones despite challenges to doing so. Studies suggest that good self-control benefits students’ task performance, positive emotions, psychological well-being, and health-promoting behaviors [28,29]. Studies have documented that self-control has a negative correlation with IGD, and poor self-control may have increased the likelihood of students developing IGD during the COVID-19 pandemic [30]. Because of unsupportive living environments, controlling relationships, and social withdrawal during the pandemic, students’ self-control was undermined, which resulted in increased IGD.

Questions regarding the correlation between social isolation and self-control and the impact of self-control on the relationship between social isolation and IGD remain largely unanswered. The limited research on this topic has reported that social isolation during the COVID-19 pandemic significantly increased the probability of internet gaming, while students with high self-control are less likely to fall into internet gaming dependence [31]. Moreover, some studies have found that self-control as a coping method is greatly associated with a variety of social isolation–related negative experiences such as anxiety, frustration, and loneliness [32,33]. According to the self-determination theory, social isolation can reduce students’ self-motivation and decision-making abilities, while maladaptive motivations (eg, introjected regulation, amotivation, strong extrinsic motivations) are positively associated with low self-control [34,35]. Low self-control was verified as an important predictor for serious IGD [36]. Therefore, social isolation may be negatively correlated with self-control, and self-control may play a mediating role in the relationship between social isolation and IGD. Students who experienced severe social isolation had poor self-control and were consequently addicted to internet gaming.

Based on these findings, this study aims to explore the associations among social isolation, self-control, and IGD and to examine the mediating effect of self-control on the relationship between social isolation and IGD in university students during the COVID-19 pandemic and related lockdown procedures in mainland China.

## Methods

### Study Participants

A cross-sectional study design was employed. University students who were Chinese and familiar with communication software (eg, WeChat, QQ) were invited to participate in this study. The surveys were conducted between April and September 2022. Convenience sampling was used to recruit university student participants from Jinan, Zibo, and Binzhou in Shandong, China. Jinan, the provincial capital of Shandong, has 23 universities. Zibo and Binzhou have 2 and 1 universities, respectively. The number of university students were 18,000-70,000. A total of 504 students completed the web-based survey. After excluding 25 questionnaires with incomplete data, 479 questionnaires were valid, with a response rate of 95.04% (479/504).

### Ethics Approval

The study survey was approved by the institutional review board of the School of Nursing and Rehabilitation, Shandong University (2021-R-043). All procedures of this study followed the guidelines and requirements of the institutional review board. An anonymous web-based survey was administered, and students who submitted the questionnaires were considered as consenting to participate in the study. Students received ¥5 (US $0.69) as compensation for their time and effort. The data were collected and stored in a computer owned by the research team, and only the researchers of this study knew the password for the data file. All procedures followed were in accordance with the ethical standards of the responsible committee on human experimentation (Shandong University, China) and with the Helsinki Declaration of 1975, as revised in 2000.

### Measurements

#### Self-Compassion Scale

The Isolation subscale of the Self-Compassion Scale [37] is a 4-item measure of social isolation. Each item contains a negative adjective description (ie, alone, inadequate, down, and struggling) to express feelings of isolation. A 5-point Likert scale from 1 (never) to 5 (always) was used to describe participants’ experience of social isolation. The Chinese version of the Self-Compassion Scale has been shown to have good validity and reliability among university students [38]. In this study, the α reliability rate for the Isolation subscale of the Self-Compassion Scale was 0.780.

#### Self-Control Scale

The Self-Control Scale, developed by Tangney et al [39], is a 36-item scale and consists of 5 dimensions: self-discipline (11 items), impulse control (10 items), healthy habits (7 items), work or study performance (4 items), and dependability (4 items). A 5-point Likert scale (1=completely out of line, 5=fully compliant) is used to score the items. The Chinese version, translated and revised by Tan and Guo [40], is a 19-item scale with 5 dimensions: impulse control (6 items), healthy habits (3 items), resisting temptation (4 items), work ethics (3 items), and abstaining from entertainment (3 items). The Chinese version also had good validity and reliability among university students, with the confirmatory factor analysis being statistically significant (*χ*^2^=1.5, root mean square error of approximation=0.050, goodness of fit index=0.91, incremental fit index=0.93, non–normed fit index=0.91, comparative fit index=0.93), and the α reliability rates for the scale and its dimension were between 0.606 and 0.862. In this study, the α reliability rates for the Self-Control Scale and its dimensions were 0.868, 0.811, 0.640, 0.648, 0.575, and 0.512, respectively.

#### IGD Scale

The IGD Scale [41] is a 9-item short scale that is scored via a 5-point Likert scale with 0 representing never and 5 representing always. The IGD Scale has been translated into Chinese, Arabic, and Turkish with good validity and reliability. The Chinese version of the IGD Scale has been widely used among young adults [42,43]. In this study, the α reliability rate for the scale was 0.888.

### Procedure

This study was conducted from April to September 2022. During this 6-month period, there was 1 outbreak of the COVID-19 pandemic in Shandong, which led to lockdown in the universities. Therefore, since students were required to maintain social distance, refrain from going outdoors, and study at home or in their dormitory, several web-based and offline methods (eg, recruitment advertisements, messages, calls) were used to recruit student participants for this study.

The Wen-Juan-Xing platform [44] was used for data collection. We created a web-based survey through Wen-Juan-Xing for business to ensure its security and functionality. The survey consisted of 2 components: an explanation and the questionnaire with 5 pages. The web-based survey link and its related QR code were sent to university counselors from 6 universities, who distributed the link and QR code to their students. Student participants were required to complete the sociodemographic questionnaire, Isolation subscale of the Self-Compassion Scale, Self-Control Scale, and IGD Scale, in that order. Each IP address can submit only 1 questionnaire. Only when the technical completeness check for the questionnaires was passed, the web-based questionnaire could be successfully submitted.

### Statistical Analysis

Harman single-factor analysis [45] was used to explore the common-method variance in this study. Descriptive analysis (mean, SD, frequency, percentage, and 95% CIs) was conducted to report the measured variables. Bivariate analysis was used to examine the differences in IGD by sociodemographic characteristics. Pearson correlation coefficients were used to test the correlations among social isolation, self-control, and IGD. Model 4 for PROCESS (Andrew F Hayes) was employed to test the mediating role of self-control on the association between social isolation and IGD. Model 5 for PROCESS was employed to explore the moderating role of gender on the association between social isolation and IGD. Bootstrap samples were set as 5000, and a 95% CI was used for the mediation analysis.

## Results

### Common-Method Variance Analysis

To avoid self-report measure–related common-method variance, we used anonymity and separating dimensions of the questionnaires in the data collection process [45]. Furthermore, Harman single-factor analysis showed that only 25.1% of the variance can be explained by the first factor, which was less than 40%. Thus, no common-method variance was found in this study.

### Descriptive Sociodemographic Characteristics and Associations Between Sociodemographic Characteristics and IGD of the Student Participants

Table 1 presents the descriptive sociodemographic characteristics of the student participants. The average age of the students was 19.97 (SD 1.81) years; 72.9% (349/479) were females, 63.5% (304/479) were not an only child, 49.3% (236/479) were freshmen, and 48.2% (231/479) were majoring in medicine. The 2-sided *t* test and analysis of variance showed that gender and major were significant influencing factors for students’ IGD (each *P*<.05).

**Table 1:** Descriptive sociodemographic characteristics of the student participants and the distribution of internet gaming disorders (N=479).

Characteristics			Values, n (%)		IGD^a^, mean (SD)		*t* test/*F* test *(df)*		*P* value
**Gender**							7.824 (3)		<.001
	Male	130 (27.1)		14.15 (9.13)					
	Female	349 (72.9)		6.99 (8.24)					
**Only child**							1.847 (3)		.07
	Yes	175 (36.5)		9.95 (9.33)					
	No	304 (63.5)		8.35 (8.86)					
**Grade**							1.334 (3, 475)		.26
	Freshman year	236 (49.3)		9.31 (8.93)					
	Sophomore year	78 (16.3)		8.67 (9.79)					
	Junior year	80 (16.7)		9.83 (8.35)					
	Senior year	85 (17.7)		7.31 (9.30)					
**Major**							5.863 (3, 475)		.001
	Liberal art	98 (20.5)		8.32 (9.29)					
	Science and Engineering	118 (24.6)		11.91 (9.50)					
	Medicine	231 (48.2)		7.81 (8.54)					
	Others	32 (6.7)		8.03 (8.31)					

^a^IGD: internet gaming disorder.

### Descriptive Social Isolation, Self-Control, and IGD and the Correlations Among the Measured Variables of Student Participants

Table 2 shows the descriptive social isolation, self-control, and IGD and the correlations among these variables of students. The total score for social isolation was 12.04 (SD 3.53), which indicated that students experienced moderate levels of social isolation during the COVID-19 pandemic. The total score for self-control was 57.15 (SD 8.44), which indicated that students had moderate levels of self-control. The total score for IGD was 8.94 (SD 9.06), which indicated that students had low levels of IGD. The Pearson correlation analysis showed that social isolation was positively correlated with IGD (*r*=0.217; *P*<.001), while social isolation and IGD were negatively correlated with self-control (*r*_social isolation_=–0.355, *r*_IGD_=–0.260; each *P*<.001).

**Table 2:** Descriptive statistics of the measured variables and the correlations among the variables (N=479).

Variables		Mean (SD)	Social isolation	Self-control	Internet gaming disorder
**Social isolation**		12.04 (3.53)	—^a^	—	—
	*r*	—	—	—	—
	*P* value	—	—	—	—
**Self-control**		57.15 (8.44)	—	—	—
	*r*	—	–0.355	—	—
	*P* value	—	<.001	—	—
**Internet gaming disorder**		8.94 (9.06)	—	—	—
	*r*	—	0.217	–0.260	—
	*P* value	—	<.001	<.001	—

^a^Not applicable.

### Mediation Analysis

Model 4 of PROCESS was employed to analyze the mediating role of self-control in the association between social isolation and IGD. Gender and major were included in the model as covariate variables. Table 3 shows the results of the mediation analysis.

Table 4 presents the effects of social isolation on IGD, which is mediated by self-control. Social isolation had a positive direct effect on IGD (β=.526; *t*=3.033; *P*<.001). When self-control as a mediator was included in the model, the positive direct effect of social isolation on IGD was also significant (β=.341; *t*=3.033; *P*=.003); moreover, social isolation had a negative direct effect on self-control (β=–.844; *t*=–8.233; *P*<.001), and self-control had a negative direct effect on IGD (β=–.219; *t*=–4.659; *P*<.001). The results showed that self-control was a significant mediator in the relationship between social isolation and IGD.

**Table 3:** Mediation model of self-control on the association between social isolation and internet gaming disorder (N=479).

Outcome variables, predictive variables			β	SE		*t* test		*P* value		*F* test *(df)*			*P* value			*R^2^*	
**Internet gaming disorder**											32.137 (4, 474)			<.001			0.163
	Social isolation	.526		.205	4.896		<.001										
	Gender	–6.995		–.344	–8.209		<.001										
	Major	–.524		–.051	–1.225		.22										
**Self-control**											23.231 (4, 474)			<.001			0.128
	Social isolation	–.844		.103	–8.233		<.001										
	Gender	–.087		.813	–0.106		.92										
	Major	–.447		.408	–1.095		.27										
**Internet gaming disorder**											30.582 (4, 474)			<.001			0.205
	Social isolation	.341		.112	3.033		.003										
	Self-control	–.219		.047	–4.659		<.001										
	Gender	–6.976		.834	–8.364		<.001										
	Major	–.426		.419	–1.016		.31										

**Table 4:** Effects of social isolation on internet gaming disorder mediated by self-control (N=479).

Effect	β (95% Boot CI)	Boot SE	Effect-total effect ratio (%)^a^
Direct effect of X^b^ on Y^c^	.341 (.120 to .562)	.112	64.83
Indirect effect: X→M1^d^→Y	–.185 (–0.295 to 0.087)	–.052	35.17

^a^Social isolation was not only directly associated with internet gaming disorder but also indirectly associated with internet gaming disorder through the path of self-control. The direct effect was 2 times stronger than the indirect effect, with effect-total effect ratios of 64.83% and 35.17%, respectively.

^b^X: social isolation.

^c^Y: internet gaming disorder.

^d^M: self-control.

### Moderation Analysis

Model 5 of PROCESS was conducted to examine whether gender mitigates the association between social isolation and IGD. Table 5 presents the moderation analysis results. The interaction variable (social isolation × gender) was negatively correlated with IGD (β=–.496; *t*=–2.003; *P*=.046), indicating that gender was a moderator in the association between social isolation and IGD.

For further analysis of the moderation of gender, a simple slope analysis was conducted. The moderation analysis of gender in the association between social isolation and IGD is depicted in Multimedia Appendix 1. The results showed that for male students, social isolation was positively correlated with IGD (β=.906; *t*=3.891; *P*<.001); for female students, social isolation was also positively correlated with IGD (β=.410; *t*=3.407; *P*=.001), but the effect size was lower than that of male students. Our results indicate that gender moderated the association between social isolation and IGD.

**Table 5:** Moderating effects of gender in the association between social isolation and internet gaming disorder, the outcome variable (N=479).

Outcome variable, predictive variables			β	SE		*t* test		*P* value		*F* test *(df)*			*R^2^*	
**Internet gaming disorder**											25.423 (5, 473)			0.212
	Self-control	–.219		.047	–4.673		<.001							
	Major	–.447		.418	–1.068		.28							
	Social isolation	.359		.112	3.198		.001							
	Gender	–6.915		.832	–8.312		<.001							
	Social isolation × gender	–.496		.248	–2.003		.046							

## Discussion

Social isolation was a crucial negative feeling experienced by university students during the COVID-19 pandemic. Since students are at a higher risk of social isolation, they develop IGD. Our study reports the associations among social isolation, self-control (a personality characteristic), and IGD and examines the mediating effect of self-control on the association between social isolation and IGD.

In our study, social isolation was found to be positively correlated with IGD, which is in line with that reported previously. Yang et al [46] suggested that because of protracted periods of social isolation during the pandemic, crisis-induced distress and technology-based activities increased greatly, thereby leading to or intensifying IGD among individuals. Social isolation is believed to disrupt students’ adaptive routines and learning habits and increase their loneliness, boredom, anxiety, frustration, and depression [47]. Due to isolation-related disruption and negative emotions, students tend to socialize on the internet platform to alleviate their stress and cater to their psychological needs. Hence, students are more likely to overuse internet games and ultimately experience IGD.

Our study shows a negative correlation between social isolation and self-control. This important finding is the first evidence, to the best of our knowledge, revealing the relationship between social isolation and self-control. Previous studies have shown that social isolation indicates individuals’ objective deficiency in interactions and support from others and the wider community, and the high prevalence of social isolation during the pandemic affected university students’ economic and social resources, coping ability, and mental health status [48]. Social isolation has been identified as a significant risk factor for emotional dysregulation and negative emotions [49,50]. Students with high levels of negative emotions tend to lose self-control in the face of difficulties and setbacks. Li et al [51] found that highly negative emotions consume individuals’ cognitive resources and impair their cognitive activities, thus decreasing their self-control capacity.

In this study, a negative correlation was found between self-control and IGD. This result corroborates those of Xiang et al [52] and Cudo et al [53], who suggested that self-control serves as a protective factor for mitigating IGD among students. Previous studies have reported that self-control represents the reasoned procedure and ability to manage impulses deliberatively [54], and students with high self-control may find it easy to withstand the temptation of internet games and to cease playing [55]. Moreover, some studies have indicated that high self-control could reduce one’s aggression and sensation-seeking attitudes, and aggressive behaviors have been related to problematic internet gaming and IGD [56].

The most interesting finding of our study was that self-control was a significant mediating factor in the association between social isolation and IGD. This important finding provides evidence for understanding the generation process of IGD among university students during a global pandemic and developing effective interventions for alleviating IGD. Because of lockdown requirements during the pandemic, university students experienced high levels of social isolation, which impacted students’ self-control [57]. Lower self-control increases the possibility of internet game playing, which eventually leads to IGD [30]. Therefore, university students become an extremely susceptible group for developing IGD. Targeted interventions to alleviate IGD should include training to promote the ability of self-control, increase alternative resources for human interactions, and remove sources of gaming temptations to reduce intentions and behaviors of playing internet games.

In this study, another interesting finding was that gender played a moderating role in the association between social isolation and IGD. Compared with male students, female students who experienced social isolation tended to have lesser IGD, indicating that male students are more vulnerable to confronting the challenges of social isolation and are more likely to play internet games to cope with isolation-related stress. Therefore, more attention should be given to male students when implementing interventions for reducing IGD.

Several limitations of this study should be considered before employing its findings. First, a cross-sectional study design was used that may hardly examine the causal relationships among the measured variables. A longitudinal or cohort study should be used instead to explore the causal effects of social isolation and self-control on IGD. Second, limited sociodemographic characteristics and personality traits (self-control) were investigated. Other personality traits (eg, self-efficacy, self-esteem, resilience, psychological capital) and family characteristics (eg, economic status, parents’ job, relationship between parents and children) should be considered in future studies. Third, convenience sampling was used to recruit student participants, which may raise some respondent bias. Random sampling is suggested to reduce these biases. Fourth, our study used the Isolation subscale of the Self-Compassion Scale to measure social isolation. Future studies are suggested to employ more complex and reliable instruments such as the Lubben Social Network Scale-6 [58] and the Orth-Gomer Measure of Social Networks Support [59]. Our study has several notable contributions to the literature on IGD in university students in mainland China. This study provides evidence of the associations among social isolation, self-control, and IGD in university students during the COVID-19 pandemic in mainland China. In particular, the negative association between social isolation and self-control was first verified. Moreover, the significant mediating effect of self-control in the relationship between social isolation and IGD was proved. Additionally, we established evidence of the impact of gender on the relationship between social isolation and IGD. In conclusion, our study emphasizes on the challenges of social isolation and IGD among university students during a pandemic and recommend that effective interventions and prevention measures be implemented with the least delay possible.

## Appendix

Multimedia Appendix 1 Moderation analysis of gender in the association between social isolation and internet game addiction.

## Abbreviations

*DSM-5*: *Diagnostic and Statistical Manual of Mental Disorders, fifth edition*
*ICD-11*: *eleventh revision of the International Classification of Diseases*
IGD: internet gaming disorder
