# Supplementary material for: COVID-19–Related Social Isolation, Self-Control, and Internet Gaming Disorder Among Chinese University Students: Cross-Sectional Survey
Source: J Med Internet Res. 2024 Sep 10;26:e52978. doi: 10.2196/52978 (PMC11422747; doi:10.2196/52978)
Supplement: Multimedia Appendix 1 [file jmir_v26i1e52978_app1.docx]

Multimedia Appendix 1 Moderation analysis of gender in the association between social isolation and internet game addiction
